# The impact of depression, stress, and self-esteem on quality of life among older adults in South Korea

**DOI:** 10.12669/pjms.40.4.8912

**Published:** 2024

**Authors:** Hye Jin Park, Yu Ra Kim, Jongsoon Choi, Sung Hee Lee, Hee Jae Kang, Hwan Ho Lee

**Affiliations:** 1 Hye Jin Park, PhD. Department of Medical Education, Eulji University School of Medicine, Daejeon, KOREA (South); 2 Yu Ra Kim, PhD. Department of Medical Humanities, Collage of Medicine, Yeungnam University, Daegu, KOREA (South); 3 Jongsoon Choi, MD., PhD. Department of Family Medicine, Kosin University College of Medicine, Busan, KOREA (South); 4 Sung Hee Lee, PhD. BRFrame Inc.Seoul, KOREA (South); 5 Hee Jae Kang, BA. BRFrame Inc.Seoul, KOREA (South); 6 Hwan Ho Lee, MD., PhD. Department of Otolaryngology-Head and Neck Surgery, Kosin University College of Medicine, Busan, KOREA (South)

**Keywords:** Elderly, Aging society, Quality of life, Depression, Stress, Self-esteem, South Korea

## Abstract

**Background & Objective:**

Aging is a global trend, and Korea is also entering an aging society, which threatens the mental health of the elderly due to isolation, etc. In line with the growing domestic and international interest in elderly issues, this study aimed to identify the effects of depression, stress and self-esteem on the lives of the elderly in South Korea and to provide basic data for welfare measures.

**Methods:**

Depression, stress, self-esteem, and quality of life were measured in 104 South Korean seniors (32 men, 72 women, average age 72.94 years old). Differences between groups according to gender and residence type were confirmed.

**Results:**

There were no significant differences in stress among the elderly by place of residence, but there were significant differences in quality of life, depression, and self-esteem. Quality of life and self-esteem were higher in private housing than in public housing, and depression was higher in public housing than in private housing. In addition, lower depression and higher self-esteem were correlated with higher quality of life among the elderly.

**Conclusion:**

With the global trend of an aging society, it is essential to continue to pay attention to assist the lives of elderly and provide them with practical support and policies. The quality of life of the elderly requires continuous attention and efforts to support and policies for mental health and economic support.

## INTRODUCTION

The aging population is increasing at an unprecedentedly fast pace in the world, putting a huge burden on Korean society^1^ and threatening the mental health of the elderly due to social isolation. In particular, the problems of mental health care such as depression and stress caused by the COVID-19 pandemic are being triggered and are expected to continue for a long time even after the pandemic ends.^2^

Life expectancy in South Korea was 77.4 years for men and 81.8 years for women, ranking 30th and 18th in the world (UN, 2007)^1^. In 2020, the life expectancy in South Korea was reported to be 83.5 years (80.5 years for men and 86.5 years for women, the healthy life expectancy, which is the life expectancy excluding the period of illness, was 66.3 years.^3^ It means if you retire at the age of 55-57, you will have about 10 years of healthy life expectancy and about 30 years of life expectancy, and if the criteria of old age is 65 or more, you will have more than 20 years to spend in old age.^2^

Researchers around the world have extensively studied quality of life and self-esteem, as key indicators of happiness, and in other words depression and stress as key indicators of unhappiness have also been studied in various ways. Depression is expressed broadly, ranging from a temporary low mood to a condition that can interfere with daily life.^4^ Depression can be a risk factor for suicide,^5^ which is why ongoing monitoring, prevention, and intervention are essential. Stress has been reported to lead to a number of health consequences,^6^ especially mental health effects such as depression.^7^ High self-esteem is a concept of feeling good about oneself, thinking competently, and evaluating oneself as a positive being,^8^ which is an important factor in maintaining health and controlling life.^9^ More than 20% of the elderly in South Korea are suffering from depression, and depression is associated with other mental health factors.^10^

Mental health has a significant impact on the quality of life of the elderly. However, there are not so many studies on the relationship between mental health and quality of life in the elderly.^1^ In particular, when looking at life satisfaction by gender, there are conflicting results, with some studies showing that elderly Male people have higher life satisfaction^11^ and others showing that elderly Female people have higher life satisfaction.^12^

In line with the growing domestic and international interest in elderly issues, this study aimed to identify the effects of depression, stress and self-esteem on the quality of life of the elderly in South Korea and to provide basic data for welfare measures.

## METHODS

### Research subjects and data collection

The scope of elderly research varies from study to study. In this study, in order to select research subjects, consent to participation in this study was sought from elderly people recruited through the South Korea Health Industry Development Institute (KHIDI)’s “Pilot project for Smart Care (Senior care medical treatment) service model demonstration”, and the study was conducted with 107 elderly people who agreed. During the research process, data from 104 people (32 men, 72 women, age average 72.94, standard deviation 5.22) were used in the final analysis, excluding three people who refused the survey or were dropped due to poor survey content. Pilot project for Smart Care (Senior care · Medical treatment) service model demonstration is an aging-friendly project that studies ways to improve the quality of life along with the physical and mental health of the elderly through smart devices such as AI. This study was conducted based on data on depression, stress, self-esteem, and quality of life in elderly people before smart care was applied.

### Research Tools

This study was conducted on older adults in South Korea using quality of life, depression, stress, and self-esteem test tools to determine the impact of depression, stress, and self-esteem on their quality of life.

### 1) Quality of life:

WHOQOL-BREF, used for quality of life testing, is a Korean version of the World Health Organization of life scale abbreviated version (WHOQOL), which is a self-reported test that assesses a people’s subjective quality of life over the past two weeks. It consists of 24 questions of the WHOQOL. The test has reported reliability (Cronbach`s α=.963)^13^ through several standardization, and the reliability (Cronbach`s α) in this study was found to be 0.908.

### 2) Depression

The Centre for Epidemiological Studies-Depression Scale (CES-D), used for depression testing, developed by Radloff et al.^14^ and adapted by Jeon et al.,^15^ consists of 20 items. Unlike other depression tests (e.g., BDI, MMPI-D, etc.), the CES-D has the advantage that it can be applied to the general population,^15^ not just patients with physical pathology. Currently, a simplified version of the test is used in the Korean Aging Research Panel Survey and the Korean Welfare Panel Survey. The reliability at the time of testing was 0.91,^15^ and Cronbach`s α of this study was reported to be 0.896.

### 3) Stress

Stress was measured by an instrument developed and modified by Cohen et al.^16^ that assesses subjects’ perceiving stress experiences over past a month on 5-point Likert scale. Each scale has been verified for reliability and validity.^17^ The total score ranges from 0 to 40, with higher scores indicating more severe perceiving stress. The reliability at the time of testing was 0.82,^18^ and Cronbach`s α of this study was reported to be 0.809.

### 4) Self-esteem

Self-esteem was measured by a tool developed by Rosenberg (1965)^19^ and utilized by the Korean Welfare Panel. Rosenberg’s constructs consist of ten questions, divided into the subconstructs of positive evaluation of oneself, self-acceptance, and self-esteem. The original scale developed by Rosenberg is a 4-point scale ranging from 0-3, but many researchers utilize a five point scale,^20^ of 1-5 in this study. In this study, the reliability (Cronbach`s α) was 0.771.

### Ethical considerations

We specify the study purpose and contents and also guarantee their anonymity. This protocol was approved by Institutional Review Board of Kosin University Gospel Hospital (IRB No. 2022-01-001-009).

### Data Analysis

The collected data was analyzed using SPSS ver. 27.0(IBM Corp., Armonk, NY, USA) in the following ways. First of all, means, standard deviations, frequencies, and percentages were calculated to identify general trends in quality of life, depression, stress, and self-esteem. In addition, independent t-tests were conducted to check for differences between groups by gender and difference in residence type, public rental housing or private housing. Next, multiple regression analysis was conducted to evaluate the effects of depression, stress, and self-esteem on quality of life Similarly, to examine the effects of depression, stress, and self-esteem on quality of life, and the differences between groups by gender and difference in residence type, public rental housing or private housing. In regression analysis, R^2^ was used to determine how much the independent variable explains the dependent variable.

## RESULTS

To test whether quality of life, depression, stress, and self-esteem differed by gender, we conducted an independent samples t-test (Table-I). The results showed significant differences in quality of life, depression, stress, and self-esteem ([p<0.01]). Quality of life and self-esteem were higher in woman elders than in man elders, however depression and stress were higher in man elders than in woman elders.

**Table-I:** Verification of variable differences by gender and place of residence.

	Total (M±SD)	Gender				Residence			

		Man (N=32) M±SD	Woman (N=72) M±SD	t	P-value	Public (N=61) M±SD	Private (N=43) M±SD	T	P-value
Quality of life	80.86±18.12	71.47±15.73	85.03±17.64	-3.74	0.00	76.82±17.73	86.58±17.29	-2.79	0.01
Depression	19.1±11.84	24.84±9.49	16.54±11.95	3.47	0.00	22.85±12.58	13.77±8.26	4.44	0.00
Stress	14.24±6.62	16.72±4.55	13.14±7.11	3.08	0.00	15.33±6.73	12.7±6.22	2.03	0.05
Self- esteem	34.91±6.79	30.22±5.93	37±6.1	-5.28	0.00	33.46±6.95	36.98±6.06	-2.68	0.01

M±SD: Mean ± Standard deviation.

Independent samples t-tests were conducted to determine if quality of life, depression, stress, and self-esteem differed by place of residence (Table-I). The results showed that only stress was not significantly different by residence, except stress, quality of life, depression, and self-esteem were significantly different. Quality of life and self-esteem were higher in private housing than in public housing(p<0.05), and depression was lower in public housing than in private housing(p<0.01).

Multiple linear regression analysis was conducted to evaluate the effect of depression, stress, and self-esteem on quality of life (Table-II). The results showed that the regression model was statistically significant (F=45.47, p<0.001), and the explanatory power of the regression model was about 57.6% (corrected R-square was 56.4%) (R^2^= .576, _adj_R^2^= .564).

**Table-II:** Effects of depression, stress, and self-esteem on quality of life.

Dependent variable	Independent variable	B	S.E	Β	t	P-value	VIF
Quality of life	(Constant)	64.200	10.300		6.233	0.00	
	Depression	-0.601	0.158	-0.393	-3.814	0.000	2.507
	Stress	-0.321	0.281	-0.117	-1.139	0.257	2.495
	Self esteem	0.937	0.227	0.351	4.127	0.000	1.707
		F=45.67 (p<0.00), R^2^=.576, _adj_R^2^=.564, D-W=2.085					

B: Unstandardized coefficient, S.E: Unstandardized coefficient standard error, β: Standardized coefficient, VIF: variance inflation factor.

According to the significance test of the regression coefficients, depression was found to have a negative effect on quality of life (β= -0.393, p< 0.001), and self-esteem was found to have a positive effect on quality of life (β= 0.351, p< 0.001). In other words, the lower depression or the higher self-esteem, the higher quality of life. Comparing the sizes of the standardized coefficients, it was verified that depression (β= -0.393), self-esteem (β= 0.351), and stress (β= -0.117) had a significant effect on the quality of life in the order (Table-II)

Multiple linear regression analysis was conducted to verify the effects of depression, stress, and self-esteem on quality of life according to gender (Table-III). The results showed that the regression model in elderly male was statistically significant (F=5.00, p< 0.01), and its explanatory power of the regression model was about 34.9% (corrected R-square was 27.9%) (R^2^=.349). The regression model in elderly Female was statistically significant (F=33.426, p< 0.01), with an explanatory power of about 59.6% (corrected R-squared was 57.8%) (R^2^=.596).

**Table-III:** Effects of depression, stress and self-esteem on quality of life by gender.

Division	Dependent variable	Independent variable	B	S.E	β	t	P-value	VIF
Old man	Quality of life	(Constant)	53.985	18.013		2.997	0.006	
		Depression	-0.181	0.354	-0.109	-0.511	0.613	1.962
		Stress	-0.732	0.724	-0.212	-1.012	0.320	1.881
		Self esteem	1.133	0.429	0.427	2.639	0.013	1.125
	F=5.000 (p<0.01), R^2^=.349, _adj_R^2^=.279, D-W=2.135							
Old woman	Quality of life	(Constant)	73.675	13.858		5.317	0.000	
		Depression	-0.742	0.176	-0.502	-4.205	0.000	2.402
		Stress	-0.259	0.310	-0.104	-0.834	0.407	2.633
		Self esteem	0.730	0.305	0.253	2.392	0.020	1.876
	F=33.426 (p<0.00), R^2^=.596, _adj_R^2^=.578, D-W=2.113							

B: Unstandardized coefficient, S.E: Unstandardized coefficient standard error, β: Standardized coefficient, VIF: variance inflation factor, D-W: Durbin-Watson.

The significance of the regression coefficient for man elderly showed that only self-esteem had a positive effect on quality of life significantly (β=.427, p<.05). In other words, the higher self-esteem of elderly Male, the higher quality of life. Comparing the sizes of the standardized coefficients, it was verified that self-esteem (β=.427), stress (β=-.212), and depression (β=-.109) had a significant effect on the quality of life in the order.

The significance test of the regression coefficients for elderly Female showed that depression had a negative effect on quality of life(β=-.502, p<.00), and self-esteem had a positive effect on quality of life (β=.253, p<.05). In other words, the lower the depression, the higher the self-esteem, the higher the quality of life.

Multiple linear regression analysis was also conducted to verify the effects of depression, stress, and self-esteem on quality of life according to residence (Table-IV). As a result, the regression model of the elderly living in public housing was statistically significant (F=45.47[24.34], p<0.001[0.01]). The explanatory power of the regression model for the elderly living in public housing was about 56.2% (corrected R squared was 53.8%) (R^2^=.576[.562]).

**Table-IV:** Effects of depression, stress and self-esteem on quality of life by place of residence

Division	Dependent variable	Independent variable	B	S.E	β	t	P-value	VIF
Living in public housing	Quality of life	(Constant)	60.202	13.164		4.573	0.000	
		Depression	-0.454	0.188	-0.322	-2.420	0.019	2.308
		Stress	-0.394	0.360	-0.150	-1.097	0.277	2.422
		Self esteem	0.988	0.290	0.387	3.402	0.001	1.683
	F=24.335 (p<0.00), R^2^=.562, _adj_R^2^=.538, D-W=2.216							
Living in his own house	Quality of life	(Constant)	71.984	16.579		4.342	0.000	
		Depression	-1.295	0.385	-0.619	-3.366	0.002	3.038
		Stress	0.202	0.507	0.073	0.400	0.692	2.982
		Self esteem	0.807	0.368	0.283	2.197	0.034	1.490
	F=16.967 (p<0.00), R^2^=.566, _adj_R^2^=.533, D-W=1.882							

B: Unstandardized coefficient, S.E: Unstandardized coefficient standard error, β: Standardized coefficient, VIF: variance inflation factor, D-W: Durbin-Watson.

The significance of the regression coefficients of the elderly living in public housing showed that self-esteem (β=.387, p<.005) had a positive effect on quality of life, and then depression had a negative effect (β= -2.420, p<.05). In other words, the higher the self-esteem [or the lower depression] of the elderly living in public housing had better quality of life. The significance test of the regression coefficients for the elderly living in private housing showed that depression had a negative effect on quality of life (β= -.619, p<.01), and on the other hand self-esteem had a positive effect on quality of life (β= .283, p<.05). In other words, the lower the depression, or the higher the self-esteem, the better the quality of life.

## DISCUSSION

The results of this study showed that depression, stress, and self-esteem affect significantly the quality of life of the elderly. Based on these findings, we make the following recommendations.

Depression had the highest negative impact on quality of life among the elderly in South Korea, followed by self-esteem, which had a positive impact on quality of life. In other words, the lower the depression or the higher the self-esteem, better the quality of life in Korean elderly people. Since depression and self-esteem are reversely corelated, and self-esteem of the elderly has a negative effect on depression,^21^ and self-esteem of women in particular is an important determinant of depression. 22 Therefore it is necessary to provide programs to prevent depression and increase self-esteem to improve the quality of life of the elderly in South Korea.

Quality of life in elderly female was better than in elderly male, and accordingly, elderly female had higher self-esteem, lower depression and less stress than elderly male, which result in depression in contrast to the finding that women were more depressed than men in college students in their 20s.^23^ In addition, the elderly are often vulnerable to stress and mental health due to physical and psychosocial changes due to aging, and this stress may cause mental health to become vulnerable.^24^,^25^ Therefore, continuous attention to depression and continuous stress management for the elderly is necessary to improve their quality of life, for which national support is needed.

Quality of life and self-esteem were higher among elderly adults living in private housing than public rental housing, and depression was lower. These results may be related to economic background. Since income satisfaction among the elderly is associated with positive self-esteem, and stable income policies are associated with lower levels of depression and stress,^24^ care, hence attention and support should be given to ensuring a stable economic conditions for the elderly.

### Limitations

This study was conducted in one city in Yeongdo-gu, Busan. Follow-up studies are needed in various regions of South Korea.

## CONCLUSION

With the global trend of aging society, the quality of life of the elderly requires continuous attention and efforts to support and State policies for mental health and economic support and essential.

### Authors Contribution:

**HJP:** Conceptualization, data curation, formal analysis, methodology, and preparing the manuscript.

**YRK:** Investigation, conceptualization, analysis, writing review, and editing.

**JSC**: Investigation, data curation, and editing.

**SHL:** Investigation, formal analysis, methodology.

**HJK**: Writing review, and editing.

**HHL:** Conceptualization, data curation, writing review & editing, final approval of the manuscript, and supervision. He is also responsible for the accuracy and integrity of the study.
